# Nonpharmaceutical Measures for Pandemic Influenza in Nonhealthcare Settings—International Travel-Related Measures 

**DOI:** 10.3201/eid2609.201990

**Published:** 2020-09

**Authors:** Jaspreet Pannu

**Affiliations:** Leland Stanford Junior University, Stanford, California, USA

**Keywords:** **:** Infrared, thermography, thermal, screening, fever, airports, viruses, influenza, coronavirus disease, severe acute respiratory syndrome coronavirus 2

**To the Editor:** Ryu et al. reviewed international travel–related measures for pandemic influenza, including screening travelers for infection (*1*). Although the authors did not review the performance of individual screening tools, Ryu et al. reported that no evidence exists to indicate that screening has any substantial effect on preventing the spread of pandemic influenza.

However, government officials continue to call for international airport screening guidelines as a crucial measure to control coronavirus disease. Therefore, differentiating between screening tools with poor technical performance and those approved for fever detection is worthwhile. For example, the Food and Drug Administration (FDA) states that thermal scanners should not be used as standalone tools for fever detection (*2*). FDA instead recommends that officials use handheld infrared thermometers as screening tools.

Thermal scanners use long-wave infrared to generate heat map images of persons and objects. This technology records surface temperature; however, fever determination requires a measurement of core body temperature. A study with 1,109 participants showed a correlation with core temperature of merely R^2^ = 0.41 for the most commonly used thermography region, the forehead (*3*). Performance of R^2^ = 0.69 was achieved only with overlaid standard camera video and complex free-form deformation models. Participants were assessed individually, after being seated for 15 minutes, without topical cosmetics or eyewear, at a stable ambient temperature and humidity, and without nearby infrared radiation sources. These conditions are rarely, if ever, met in the airport setting.

Despite this evidence, costly thermal scanners have been deployed at airports in many countries. In contrast, inexpensive infrared thermometers are FDA approved for core temperature approximation. At their current performance, thermal scanners must be clearly distinguished from infrared thermometers, and thermal scanning should not be recommended for fever screening.
